# Fluoxetine and Sertraline Potently Neutralize the Replication of Distinct SARS-CoV-2 Variants

**DOI:** 10.3390/v16040545

**Published:** 2024-03-30

**Authors:** Laura Thümmler, Nadine Beckmann, Carolin Sehl, Matthias Soddemann, Peer Braß, Maren Bormann, Leonie Brochhagen, Carina Elsner, Nicolas Hoertel, Céline Cougoule, Sandra Ciesek, Marek Widera, Ulf Dittmer, Monika Lindemann, Peter A. Horn, Oliver Witzke, Stephanie Kadow, Markus Kamler, Erich Gulbins, Katrin Anne Becker, Adalbert Krawczyk

**Affiliations:** 1 Department of Infectious Diseases, West German Centre of Infectious Diseases, University Medicine Essen, University Hospital Essen, University Duisburg-Essen, 45147 Essen, Germany; laura.thuemmler@uk-essen.de (L.T.); peer.brass@uk-essen.de (P.B.); maren.bormann@uk-essen.de (M.B.); leonie.brochhagen@uk-essen.de (L.B.); oliver.witzke@uk-essen.de (O.W.); 2 Institute for Transfusion Medicine, University Hospital Essen, University Duisburg-Essen, 45147 Essen, Germany; monika.lindemann@uk-essen.de (M.L.); peter.horn@uk-essen.de (P.A.H.); 3 Institute of Molecular Biology, University Hospital Essen, University of Duisburg-Essen, 45147 Essen, Germanycarolin.sehl@uk-essen.de (C.S.); matthias.soddemann@uk-essen.de (M.S.); stephanie.kadow@uk-essen.de (S.K.); erich.gulbins@uk-essen.de (E.G.); katrin.becker-flegler@uni-due.de (K.A.B.); 4 Institute for Virology, University Hospital Essen, University Duisburg-Essen, 45147 Essen, Germany; carina.elsner@uk-essen.de (C.E.); ulf.dittmer@uk-essen.de (U.D.); 5 Institute Psychiatry and Neuroscience de Paris, INSERM U1266, Paris Cité University, 75014 Paris, France; nicolas.hoertel@aphp.fr; 6 Psychiatry and Addiction Department Corentin-Celton Hospital (AP-HP), 92130 Paris, France; 7 Institute of Pharmacology and Structural Biology (IPBS), CNRS, University of Toulouse, UPS, 31000 Toulouse, France; celine.cougoule@ipbs.fr; 8 Institute of Medical Virology, University Hospital Frankfurt, 60590 Frankfurt am Main, Germany; sandra.ciesek@ukffm.de (S.C.); marek.widera@ukffm.de (M.W.); 9 Institute of Pharmaceutical Biology, Goethe-University, 60323 Frankfurt am Main, Germany; 10 Fraunhofer Institute for Molecular Biology and Applied Ecology (IME), Branch Translational Medicine and Pharmacology, 60311 Frankfurt am Main, Germany; 11 Department of Thoracic and Cardiovascular Surgery, West German Heart Center, University Hospital Essen, 45147 Essen, Germany; markus.kamler@uk-essen.de

**Keywords:** SARS-CoV-2, antidepressants, COVID-19

## Abstract

The pandemic caused by SARS-CoV-2 is still a major health problem. Newly emerging variants and long-COVID-19 represent a challenge for the global health system. In particular, individuals in developing countries with insufficient health care need easily accessible, affordable and effective treatments of COVID-19. Previous studies have demonstrated the efficacy of functional inhibitors of acid sphingomyelinase against infections with various viruses, including early variants of SARS-CoV-2. This work investigated whether the acid sphingomyelinase inhibitors fluoxetine and sertraline, usually used as antidepressant molecules in clinical practice, can inhibit the replication of the former and recently emerged SARS-CoV-2 variants in vitro. Fluoxetine and sertraline potently inhibited the infection with pseudotyped virus-like particles and SARS-CoV-2 variants D614G, alpha, delta, omicron BA.1 and omicron BA.5. These results highlight fluoxetine and sertraline as priority candidates for large-scale phase 3 clinical trials at different stages of SARS-CoV-2 infections, either alone or in combination with other medications.

## 1. Introduction

Infections with severe acute respiratory syndrome coronavirus-2 (SARS-CoV-2) have declined because of successful vaccination campaigns and the lower lethality of omicron variants compared to the former ones, and hospitalization rates have dropped significantly. Nevertheless, the virus is still responsible for more than 20,000,000 current infections, including severe and life-threatening diseases [1]. In low- and middle-income countries worldwide, coronavirus disease 2019 (COVID-19) remains a serious health problem [2]. Although several vaccines have been approved within an impressively short time, public opposition against vaccination and an increasing vaccine fatigue are major obstacles in maintaining immunity in the worldwide population [3]. Furthermore, newly emerging SARS-CoV-2 variants accompanied by an increasing number of breakthrough infections require developing prophylactic and therapeutic treatment options against SARS-CoV-2 infections [4,5,6]. Antidepressants represent a promising class of cheap and globally available drugs with proven antiviral activity against SARS-CoV-2 [7].

Several pre-clinical and clinical studies demonstrated that antidepressants belonging to the group of functional inhibitors of the acid sphingomyelinase (FIASMA) showed a broad antiviral activity against viruses such as enteroviruses [8,9,10], coxsackieviruses [11], hepatitis C [12], arenaviruses [13] and SARS-CoV-2 [7,14,15,16,17,18,19]. Notably, patients treated with such antidepressants showed less severe COVID-19 symptoms, a less frequent requirement of ventilation and most importantly showed a less often lethal course of disease [20,21,22,23].

We have previously demonstrated that pre-treatment of cultured Vero E6 epithelial cells or freshly isolated human nasal epithelial cells with antidepressants such as amitriptyline, desipramine, imipramine, fluoxetine, sertraline, maprotiline or escitalopram prevented infections with SARS-CoV-2 spike pseudotyped vesicular stomatitis virus (VSV) particles and an early isolate of SARS-CoV-2 D614G [24].

Mechanistically, we demonstrated that binding of SARS-CoV-2 spike to ACE2, the cellular receptor of SARS-CoV-2, induces activation of the acid sphingomyelinase, the subsequent conversion of sphingomyelin to ceramide and the formation of ceramide-enriched membrane platforms at the outer leaflet of the plasma membrane [24,25]. Inhibition of the acid sphingomyelinase by antidepressants prevented these biochemical changes and therefore blocked the infection of target cells with SARS-CoV-2 [24].

Thus, antidepressants targeting the acid sphingomyelinase represent a promising strategy for the treatment and prevention of severe COVID-19 [26,27]. Although initial studies demonstrated potent antiviral activity against SARS-CoV-2 D614G, it remains unclear if antidepressants such as fluoxetine and sertraline can interfere with the replication of newly emerged SARS-CoV-2 variants of concern.

In the present study, we investigated the antiviral activity of fluoxetine and sertraline against the SARS-CoV-2 variants D614G, alpha, delta, omicron BA.1 and omicron BA.5.

## 2. Materials and Methods

### 2.1. Cells and Viruses

All cell lines were incubated at 37 °C and 5% CO_2_ in a humidified atmosphere in a standard tissue incubator unless specifically indicated otherwise. HEK293T (human kidney cell line) cells were obtained from Merck (Darmstadt, Germany) via the lab of Ulf Brockmeier (Department of Neurology and Center for Translational Neuro- and Behavioral Sciences (C-TNBS), University Hospital Essen, Essen, Germany) and maintained in Dulbecco’s modified Eagle medium (DMEM; Thermo Fisher Scientific, Waltham, MA, USA) supplemented with 10% fetal bovine serum (FBS) (GE Healthcare, Chicago, IL, USA), 100 U/mL penicillin and 0.1 mg/mL streptomycin (Thermo Fisher Scientific). HEK293T cells were used for the production of VSV*∆G-Fluc spike pseudotype virus-like particles. Vero E6 (African green monkey kidney cell line) cells were obtained from ATCC and maintained in DMEM supplemented with 10% FBS (GE Healthcare), 100 U/mL penicillin, 0.1 mg/mL streptomycin, 100 µM non-essential amino acids, 1 mM sodium pyruvate (each Thermo Fisher Scientific) and 10 mM HEPES, pH 7.3 (Carl Roth, Karlsruhe, Germany). Vero cells were utilized in the preliminary infection experiments with pseudotype virus-like particles. Human lung A549-AT cells stably transfected with ACE2 and TMPRSS2 [28] were cultured in minimal essential medium (MEM, Thermo Fisher Scientific) supplemented with 10% FBS, 100 U/mL penicillin, 0.1 mg/mL streptomycin, 100 µM non-essential amino acids, 1 mM sodium pyruvate and 10 mM HEPES, pH 7.3 (each Thermo Fisher Scientific). The enhanced expression of ACE2 and TMPRSS2 contributed to a robust infection rate of cells with various SARS-CoV-2 variants. This justified the utilization of these cells for evaluating the antiviral efficacy of fluoxetine and sertraline against different clinical SARS-CoV-2 isolates.

The clinical SARS-CoV-2 isolates of wild type (D614G), alpha, delta, omicron BA.1 and BA.5 were obtained from nasopharyngeal swabs of COVID-19 patients hospitalized at the University Hospital Essen, Germany, as previously described [4,29]. The SARS-CoV-2 variants were identified after sequencing the spike gene and correlating with the corresponding variants according to the list of variants of concern from the WHO [World Health Organization Tracking SARS-CoV-2 Variants [30]]. The viruses were propagated on A549-AT cells and stored at −80 °C. Viral titers were determined by a standard endpoint dilution assay and calculated as 50% tissue culture infective dose (TCID_50_)/mL, as previously described [31]. The clinical SARS-CoV-2 isolates used here were sequenced in a previous work [4]. The datasets presented in this study can be found online (ENA; https://www.ebi.ac.uk/ena/browser/view/PRJEB59607 (accessed on 9 January 2024)).

### 2.2. Plasmids

The initial neutralization experiments were performed using pp-VSV-SARS-CoV-2 spike pseudotyped virus-like particles. These were generated by using pCG1_SARS-2-S-del18 plasmid, which was kindly provided by Markus Hoffmann and Stefan Pöhlmann (Infection Biology Unit, German Primate Centre–Leibniz Institute for Primate Research, Göttingen, Germany) and previously described [32]. SARS-CoV-2 spike variants with point mutations in the receptor-binding domain (RBD) were generated sequentially by using the Q5 site-directed mutagenesis kit (New England Biolabs, Ipswich, MA, USA) according to the manufacturer’s instructions and confirmed by sequencing (Table S1).

Expression plasmids carrying all mutations of the respective variants of concern were a gift from David Nemazee (Wt: pCDNA3.3_CoV2_D18, Addgene plasmid #170442; alpha: pCDNA3.3_CoV2_B.1.1.7, Addgene plasmid #170451; beta: pCDNA3.3_CoV2_501V2, Addgene plasmid #170449; gamma: pCDNA3.3_CoV2_P1, Addgene plasmid number #170450; delta: pCDNA3.3-SARS2-B.1.617.2, Addgene plasmid #172320) or obtained from GenScript (omicron: SARS-CoV-2 Omicron Strain S gene Human codon_pcDNA3.1(+), #MC_0101274) (Table S2). Plasmids were amplified in *E. coli* DH5α competent cells (New England Biolabs) and isolated and purified using Maxiprep Kits according to the manufacturer’s instructions (Qiagen, Hilden, Germany and Macherey-Nagel, Düren, Germany).

### 2.3. Preparation of VSV*∆G-Fluc Spike Pseudotype Virus-like Particles

The replication-restricted VSV system for pseudoviral particles with enhanced green fluorescent protein (eGFP) and firefly luciferase (Fluc) reporters (VSV*∆G-FLuc) was kindly provided by Gert Zimmer (Institute of Virology and Immunology, Mittelhäusern/Switzerland), passed on by the lab of Stefan Pöhlman and previously described by Berger Rensch and Zimmer [33]. We followed the protocol for pseudoviral particle generation described by Becker et al. [34]. VSV*ΔG-Fluc (VSV-G) stocks and anti-VSV-G antibody supernatants were prepared and titrated as described. The SARS-CoV-2 pseudotyped VSV particles were prepared according to the described protocol, but with slight modifications; low-passage HEK293T cells were seeded into 10 cm cell culture dishes (at 1.8 × 10^6^ cells/dish) and grown for 24 h at 37 °C with 5% CO_2_ in a standard cell culture incubator. On the next day, the cells received fresh medium (9 mL/dish), and the transfection mixtures were prepared by mixing 42 µg of the respective plasmid DNA per dish and sterile ultrapure water to a final volume of 400 µL. A total of 100 µL sterile-filtered CaCl_2_ (stock conc. 2.5 M, Thermo Fisher Scientific) was added and mixed. A total of 500 µL 2× HBS buffer (280 mM NaCl, 50 mM HEPES, 20 mM KCl, 1.5 mM Na_2_HPO_4_, pH 7.1, each Thermo Fisher Scientific) was added dropwise while bubbling the solution with an electronic pipettor. The mixture was then immediately vortexed and incubated for 20 min at room temperature. The transfection complexes were added dropwise to the cells and incubated for 28-30 h at 33 °C with 5% CO_2_. Cells were inoculated with VSV*ΔG-FLuc at a multiplicity of infection of five for one hour at 33 °C and 5% CO_2_ in a standard tissue incubator. The supernatant was then removed and cells washed two times with PBS (Thermo Fisher Scientific). Fresh medium containing anti-VSV-G antibodies was added, and cells were incubated overnight (18 h) at 33 °C with 5% CO_2_ in a standard tissue incubator. The supernatant containing the pseudoviral particles was then harvested, cellular debris was removed by centrifugation at 2000× *g* for 10 min and the clarified supernatant was used immediately for experiments.

### 2.4. Transduction In Vitro Experiments

Vero E6 cells were seeded at 1 × 10^4^ cells/well in 48-well plates and grown for 48 h. Cells were then pre-treated for 4 h with antidepressants in DMEM (Thermo Fisher Scientific) with only 1% FBS (Thermo Fisher Scientific) prior to infection. Fluoxetine hydrochloride and sertraline hydrochloride (each Sigma Aldrich, St. Louis, MO, USA) were dissolved in dimethylsulfoxide (DMSO, Carl Roth) and diluted to a final concentration of 25 µM fluoxetine or 10 µM sertraline. Mock treated cells were assessed as controls. The medium was removed, and the cells were incubated with a medium containing VSV*ΔG-Fluc spike and 25 µM fluoxetine or 10 µM sertraline. The medium containing VSV*∆G-Fluc spike and corresponding DMSO concentrations without the addition of antidepressants served as the control. Cells were infected for 1 h at 33 °C with 5% CO_2_ in a standard tissue culture incubator. Pseudoviral supernatants were discarded, and the cells overlaid with fresh medium and incubated for 18 h at 33 °C with 5% CO_2_. The effect of antidepressants on the infection of the cells was assessed by qualitative and quantitative analysis of the eGFP expression. The cell cultures were scanned for eGFP fluorescence on a Typhoon FLA biomolecular imager (GE Healthcare) and microscopic fluorescence images were obtained (EVOS, Thermo Fisher Scientific). For quantitative analysis, cells were harvested for flow cytometric analysis (Attune NxT, Thermo Fisher Scientific). The infection efficacy in the mock controls was set as 100%, and the effect of the antidepressants was reported relative to these controls.

### 2.5. Neutralization Assay

The antiviral activity of fluoxetine and sertraline was determined by a cell-culture-based endpoint dilution assay. A549-AT cells were seeded in a 24-well plate (at 1 × 10^4^ cells/well) and grown for 24 h at 37 °C with 5% CO_2_ in a standard cell culture incubator. The confluent A549-AT cells were pre-incubated with different concentrations of fluoxetine (0 µM, 5 µM, 10 µM, 20 µM, 25 µM or 30 µM) or sertraline (0 µM, 5 µM or 10 µM) with MEM (Thermo Fisher Scientific) supplemented with 2% FBS, 100 U/mL penicillin, 0.1 mg/mL streptomycin, 100 µM non-essential amino acids, 1 mM sodium pyruvate and 10 mM HEPES, pH 7.3 (each Thermo Fisher Scientific) for two hours at 37 °C with 5% CO_2_. Subsequently, the A549-AT cells were infected by adding 100 TCID_50_ of SARS-CoV-2 into the medium, and the cells were then incubated for three days at 37 °C with 5% CO_2_. Untreated A549-AT cells served as a negative control. Thereafter, the supernatants were harvested and the viral titers were determined by endpoint dilution. Therefore, serial dilutions of the cell culture media (1:10–1:10^8^) were incubated on A549-AT cells grown on a 96-well microtiter plate for three days at 37 °C with 5% CO_2_. The inoculation medium was then removed, the cells stained with 0.5% crystal violet (Roth), solved in 20% methanol (Merck) and evaluated for cytopathic effects (CPEs) using light microscopy. The experiment was performed in triplicate.

### 2.6. Quantification of SARS-CoV-2 RNA

Cell culture supernatants were centrifuged to remove cell debris and stored at −80 °C before further processing. Total RNA was purified from cell culture supernatants using the QIAmp viral RNA Mini Kit (Qiagen) according to manufacturer’s instructions with preceding DNase I digestion with the RNase-Free DNase Set (Qiagen). A total of 250 ng of total RNA was reverse transcribed using the PrimeScript RT Master Mix (Takara, Kusatsu, Japan) for relative determination of SARS-CoV-2 M- or N-gene expression. For RT-qPCR, GoTaq Probe qPCR Master Mix (Promega, Madison, WI, USA) was used according to the manufacturer’s instructions, with gene-specific primers and probes (Table S3). RT-qPCR was performed on a LightCycler 480 II (Roche, Basel, Switzerland) instrument, with the following conditions: initial denaturation for 2 min at 95 °C with a ramp rate of 4.4 °C/s, followed by 40 cycles of denaturation for 15 s at 95 °C with a ramp rate of 4.4 °C/s and amplification for 60 s at 60 °C with a ramp rate of 2.2 °C/s. To assess M- and N-gene copy numbers, the M- and N-genes were partially cloned into pCR2.1 (Thermo Fisher Scientific) or pMiniT 2.0 (NEB, Ipswich, MA, USA), respectively. A 1:10 plasmid dilution series was used as a reference.

### 2.7. Cytotoxicity Assay

Potential cytotoxicity of various concentrations of fluoxetine and sertraline towards A549-AT cells was determined using the Orangu™ cell counting solution (Cell guidance systems, Cambridge, UK) according to the manufacturer’s protocol. Briefly, increasing fluoxetine or sertraline concentrations (0 µM, 5 µM, 10 µM, 20 µM or 30 µM) in MEM (Thermo Fisher Scientific) were supplemented with 2% FBS, 100 U/mL penicillin, 0.1 mg/mL streptomycin, 100 µM non-essential amino acids, 1 mM sodium pyruvate and 10 mM HEPES, pH 7.3 (each Thermo Fisher Scientific) and incubated with 1 × 10^4^ A549-AT cells per well of a 96-well-plate at 37 °C, 5% CO_2_. At four distinct time points (2 h, 24 h, 48 h and 72 h), the inoculation medium was removed and replaced by a medium containing 10% of Orangu™ cell counting solution. After 60 min of incubation (37 °C, 5% CO_2_), cell viability was measured at an absorbance of 450 nm using Tristar 3 (Berthold Technologies, Oak Ridge, TN, USA) and normalized to untreated control cells. The experiment was performed in triplicate.

### 2.8. Statistics

Statistical analysis was performed using GraphPad Prism 10.0 software (San Diego, CA, USA). The analysis of categorical variables was performed by one-way ANOVA (Friedman test) with Dunnett’s correction for multiple comparisons, as appropriate. Two-sided *p* values < 0.05 were considered significant.

## 3. Results

### 3.1. Initial Investigation of the Antiviral Activity of Fluoxetine and Sertraline against SARS-CoV-2

As the first step, the ability of fluoxetine and sertraline to inhibit the SARS-CoV-2 infection of cells was evaluated with VSV*∆G-Fluc spike pseudotyped virus-like particles. Therefore, VeroE6 cells were pre-treated for 4 h with 25 µM fluoxetine or 10 µM sertraline and subsequently infected with different VSV*∆G-Fluc spike variant pseudotyped virus-like particles for 1 h, and the antiviral efficiency was assessed by fluorescence microscopy as well as quantitatively by flow cytometry the next day.

Initially, VSV*∆G-Fluc spike pseudotyped virus-like particles with mutations only in the spike RBD region were used. Fluoxetine treatment led to significantly lower infection rates with wild type (*p* < 0.0001), alpha (*p* < 0.0001), beta (*p* < 0.0001), gamma (*p* < 0.0001), delta (*p* < 0.0001), lambda (*p* < 0.0001) and mu (*p* < 0.0001) compared to the DMSO control. The mean percentage infection of cells treated with fluoxetine was 48% for wild type, 43% for alpha, 35% for beta, 42% for delta, 35% for gamma, 58% for lambda and 66% for mu.

**Figure 1:**
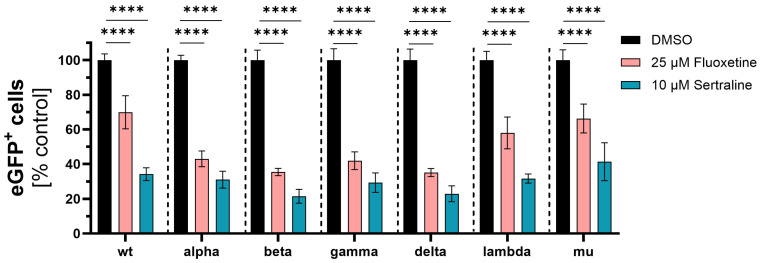
Inhibition of SARS-CoV-2 infection with distinct VSV*∆G-Fluc spike pseudotyped virus-like particles. Vero E6 cells were pre-treated with either DMSO, 25 µM fluoxetine or 10 µM sertraline and subsequently infected with VSV*∆G-Fluc spike pseudotyped virus-like particles with RBD mutations of different SARS-CoV-2 variants. The effect of antidepressants on the infection of the cells was assessed by quantitative analysis of the eGFP expression. Data are shown as mean and standard deviation (SD). Differences between fluoxetine/sertraline and the DMSO control were compared by one-way ANOVA (**** *p* < 0.0001).

Accordingly, treatment with sertraline led to significantly lower infection rates with wild type (*p* < 0.0001), alpha (*p* < 0.0001), beta (*p* < 0.0001), gamma (*p* < 0.0001), delta (*p* < 0.0001), lambda (*p* < 0.0001) and mu (*p* < 0.0001). In the case of sertraline treatment, the mean percentage infection of cells observed was 37% for wild type, 31% for alpha, 21% for beta, 29% for gamma, 23% for delta, 32% for lambda and 41% for mu. Overall, sertraline treatment resulted in a greater reduction of infection compared to fluoxetine (Figure 1).

**Figure 2:**
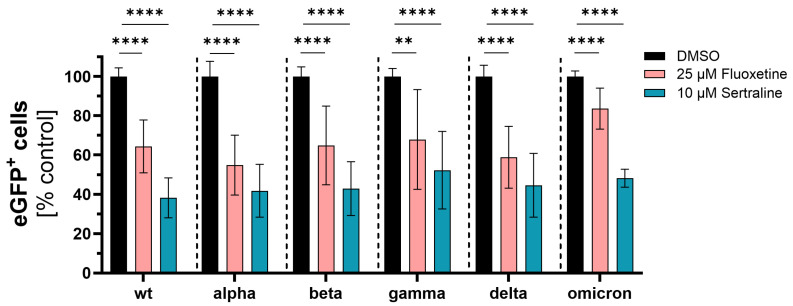
Inhibition of SARS-CoV-2 infection with distinct commercial VSV*ΔG-Fluc spike pseudotype virus like particles. Vero E6 cells were pre-treated with either DMSO, 25 µM fluoxetine or 10 µM sertraline and subsequently infected with VSV*ΔG-Fluc spike pseudotyped virus-like particles with mutations of different SARS-CoV-2 variants. The effect of antidepressants on the infection of the cells was assessed by quantitative analysis of the eGFP expression. Data are shown as mean and standard deviation (SD). Differences between fluoxetine/sertraline and the DMSO control were compared by one-way ANOVA (** *p* < 0.01; **** *p* < 0.0001).

Next, expression plasmids coding for spike proteins containing all mutations of the respective variants of concern were used to generate VSV*∆G-Fluc pseudotyped virus-like particles, and the inhibitory effect of fluoxetine and sertraline against the infection of cells with SARS-CoV-2 was analyzed. Fluoxetine significantly reduced the infection of the cells with VSV*∆G-Fluc spike pseudotyped virus-like particles of wild type D614G to 64% (*p* < 0.0001), of alpha to 55% (*p* < 0.0001), of beta to 65% (*p* < 0.0001), of gamma to 68% (*p* = 0.0025), of delta to 59% (*p* < 0.0001) and of omicron to 84% (*p* < 0.0001) compared to the DMSO control. Sertraline also led to a significant reduction of the infection with VSV*∆G-Fluc spike pseudotyped virus-like particles of wild type D614G to 38% (*p* < 0.0001), alpha to 42% (*p* < 0.0001), beta to 43% (*p* < 0.0001), gamma to 52% (*p* < 0.0001), delta to 45% (*p* < 0.0001) and omicron to 48% (*p* < 0.0001) compared to the DMSO control. Fluoxetine and sertraline showed broad antiviral activity against VSV*ΔG-Fluc spike pseudotyped virus-like particles displaying distinct SARS-CoV-2 spike protein variants. Interestingly, sertraline more potently inhibited the infection with VSV*ΔG-Fluc spike pseudotyped virus-like particles compared to fluoxetine (Figure 2).

**Figure 3:**
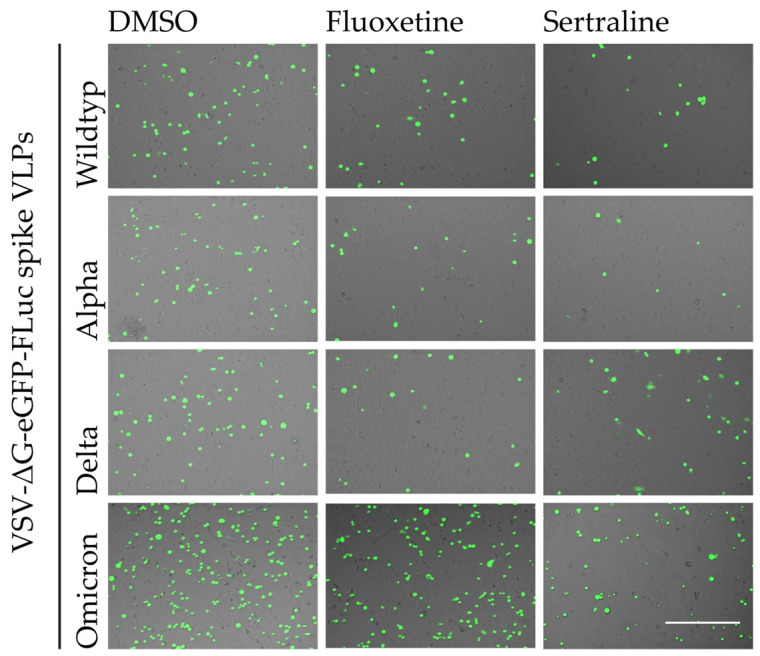
Qualitative assessment of the antiviral activity of fluoxetine and sertraline against SARS-CoV-2. Vero E6 cells were pre-treated with either DMSO, 25 µM fluoxetine or 10 µM sertraline and subsequently infected with VSV*ΔG-Fluc spike pseudotyped virus-like particles with mutations of different SARS-CoV-2 variants. The effect of antidepressants on the infection of the cells was assessed by fluorescence microscopy. Scale bar represents 400 µm.

The inhibitory effect of fluoxetine and sertraline on the SARS-CoV-2 infection of Vero E6 cells with different VSV*ΔG-Fluc spike pseudotype virus-like particles was confirmed by fluorescence microscopy (Figure 3).

### 3.2. Fluoxetine and Sertraline Effectively Blocks SARS-CoV-2 Infection

To examine whether the antidepressants fluoxetine and sertraline can inhibit infection with a real virus, the antiviral efficacy of fluoxetine (0–30 µM) or sertraline (0–10 µM) was investigated against clinical isolates SARS-CoV-2 D614G, alpha, delta, omicron BA.1 and omicron BA.5.

**Figure 4:**
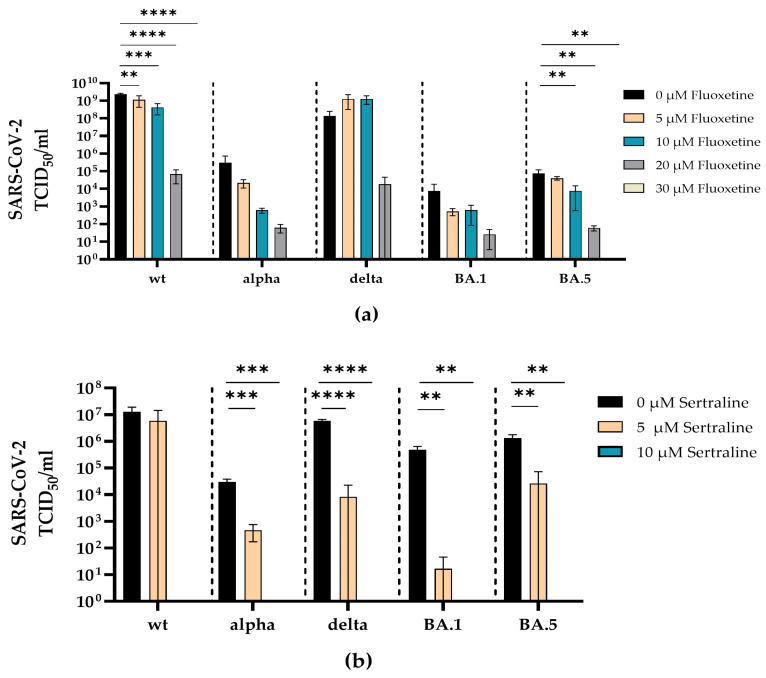
Fluoxetine and sertraline completely inhibit SARS-CoV-2 infection. A549-AT cells were infected with 100 TCID_50_ of different clinical SARS-CoV-2 isolates (D614G, alpha, delta, omicron BA.1 and omicron BA.5) in the presence of different concentrations of fluoxetine (0–30 µM) (**a**) or sertraline (0–10 µM) (**b**). After three days of incubation, the viral loads in the respective cell culture supernatants were determined by endpoint dilution. The experiments were performed in triplicate. Data are shown as mean and standard deviation (SD). Differences between fluoxetine/sertraline and the control were compared by one-way ANOVA (** *p* < 0.01; *** *p* < 0.001; **** *p* < 0.0001).

Fluoxetine inhibited the replication of SARS-CoV-2 D614G, alpha, delta, omicron BA.1 and omicron BA.5 in a dose-dependent manner. Complete inhibition of all SARS-CoV-2 variants was reached at a concentration of 30 µM. Significant reduction of the viral load was found for SARS-CoV-2 D614G (5 µM *p* = 0.0097; 10 µM *p* = 0.0003; 20 µM *p* < 0.0001; 30 µM *p* < 0.0001) and omicron BA.5 (10 µM *p* = 0.0073; 20 µM *p* = 0.0037; 30 µM *p* = 0.0036) compared to the medium control (Figure 4a).

Accordingly, sertraline inhibited the replication of SARS-CoV-2 D614G, alpha, delta, omicron BA.1 and omicron BA5 in a dose-dependent manner, where all SARS-CoV-2 variants were completely neutralized at a concentration of 10 µM sertraline. The incubation with sertraline significantly decreased the viral replication of SARS-CoV-2 alpha (5 µM *p* = 0.0006; 10 µM *p* = 0.0005), delta (5 µM *p* < 0.0001; 10 µM *p* < 0.0001), omicron BA.1 (5 µM *p* = 0.0012; 10 µM *p* = 0.0012) and omicron BA.5 (5 µM *p* = 0.0017; 10 µM *p* = 0.0016) at both tested concentrations compared to the control (Figure 4b).

**Figure 5:**
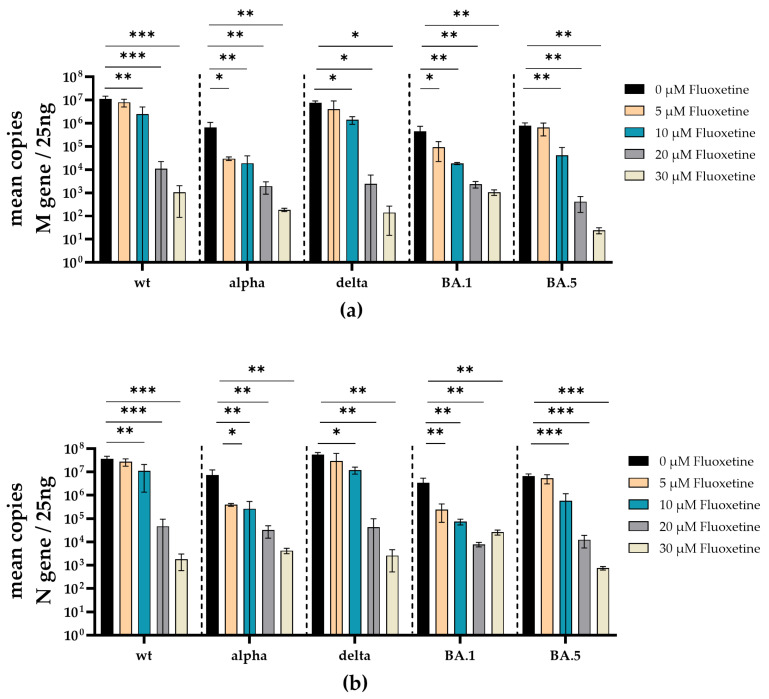
Treatment with fluoxetine significantly reduces SARS-CoV-2 M- and N-gene levels. A549-AT cells were infected with 100 TCID_50_ of different SARS-CoV-2 variants in the presence of different concentrations of fluoxetine (0–30 µM). After incubation for 72 h, supernatants were harvested. Total RNA was isolated and reverse transcribed, and the SARS-CoV-2 M- (**a**) and N-gene (**b**) were quantified by RT-qPCR. Data are shown as mean and standard deviation (SD). Differences between fluoxetine/sertraline and the control were compared by one-way ANOVA (* *p* < 0.05; ** *p* < 0.01; *** *p* < 0.001).

To additionally validate the antiviral effect of fluoxetine and sertraline against SARS-CoV-2 variants, SARS-CoV-2 RNA from cell culture supernatants of infected and fluoxetine- or sertraline-treated cells was quantified. Therefore, the RNA of the respective supernatant was isolated and reverse transcribed, and the M- and N-gene of SARS-CoV-2 were quantified by RT-qPCR. Fluoxetine treatment reduced the copy-number of SARS-CoV-2 M- and N-gene RNA in a dose-dependent manner. There was a significant reduction of M-gene RNA of SARS-CoV-2 D614G (10 µM *p* = 0.0039; 20 µM *p* = 0.0006; 30 µM *p* = 0.0006), alpha (5 µM *p* = 0.0107; 10 µM *p* = 0.0096; 20 µM *p* = 0.0078; 30 µM *p* = 0.0076), delta (10 µM *p* = 0.037; 20 µM *p* = 0.011; 30 µM *p* = 0.011), omicron BA.1 (5 µM *p* = 0.029; 10 µM *p* = 0.0099; 20 µM *p* = 0.0079; 30 µM *p* = 0.0077) and omicron BA.5 (10 µM *p* = 0.0032; 20 µM *p* = 0.0022; 30 µM *p* = 0.0022) at different fluoxetine concentrations (Figure 5a). Furthermore, a significant reduction of N-gene RNA was found in cell culture supernatants from SARS-CoV-2 D614G (10 µM *p* = 0.0085; 20 µM *p* = 0.0006; 30 µM *p* = 0.0006), alpha (5 µM *p* = 0.010; 10 µM *p* = 0.0096; 20 µM *p* = 0.0078; 30 µM *p* = 0.0076), delta (10 µM *p* = 0.024; 20 µM *p* = 0.0056; 30 µM *p* = 0.0056), omicron BA.1 (5 µM *p* = 0.0043; 10 µM *p* = 0.0030; 20 µM *p* = 0.0026; 30 µM *p* = 0.0027) and omicron BA.5 (10 µM *p* = 0.0005; 20 µM *p* = 0.0003; 30 µM *p* = 0.0003) infected cells treated with fluoxetine (Figure 5b). The M-gene RNA decreased slightly more than the N-gene RNA after treatment with fluoxetine.

**Figure 6:**
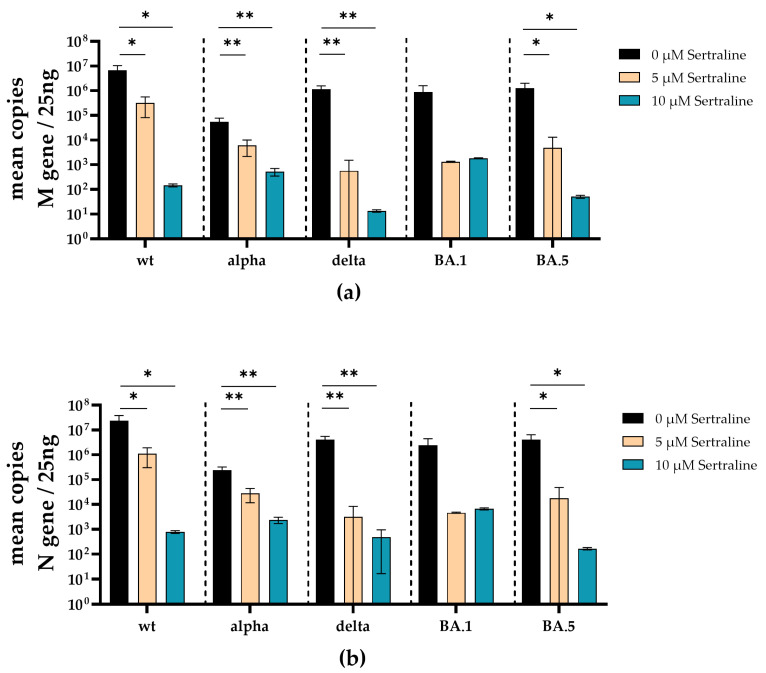
Treatment with sertraline significantly reduces SARS-CoV-2 M- and N-gene levels. A549-AT cells were infected with 100 TCID_50_ of different SARS-CoV-2 variants in the presence of different sertraline concentrations (0–10 µM). After 72 h of incubation, the supernatants were harvested and the total RNA was isolated and reverse transcribed, and the M- (**a**) and N-gene (**b**) of SARS-CoV-2 were quantified by RT-qPCR. Data are shown as mean and standard deviation (SD). Differences between fluoxetine/sertraline and the control were compared by one-way ANOVA (* *p* < 0.05; ** *p* < 0.01).

Similar to the treatment with fluoxetine, the total amounts of SARS-CoV-2 M- and N-gene RNA were substantially decreased under sertraline treatment. Significant reduction of M-gene RNA was found in the supernatants of SARS-CoV-2 D614G (5 µM *p* = 0.021; 10 µM *p* = 0.017), alpha (5 µM *p* = 0.0052; 10 µM *p* = 0.0030), delta (5 µM *p* = 0.0028; 10 µM *p* = 0.0028) and omicron BA.5 (5 µM *p* = 0.0173; 10 µM *p* = 0.0170) infected and sertraline-treated cells. SARS-CoV-2 N-gene RNA levels of omicron BA.1 were reduced by several log levels but did not reach a significant level (Figure 6a). Furthermore, a significant decrease in N-gene RNA was found in cell culture supernatants from SARS-CoV-2 D614G (5 µM *p* = 0.027; 10 µM *p* = 0.022), alpha (5 µM *p* = 0.0040; 10 µM *p* = 0.0022), delta (5 µM *p* = 0.0016; 10 µM *p* = 0.0016) and omicron BA.5 (5 µM *p* = 0.0209; 10 µM *p* = 0.0205) infected cells treated with sertraline. Moreover, SARS-CoV-2 N-gene RNA levels of omicron BA.1 were reduced by several log levels but did not reach a significant level (Figure 6b).

### 3.3. Fluoxetine and Sertraline Showed No Toxic Effects at SARS-CoV-2 Neutralizing Concentrations

**Figure 7:**
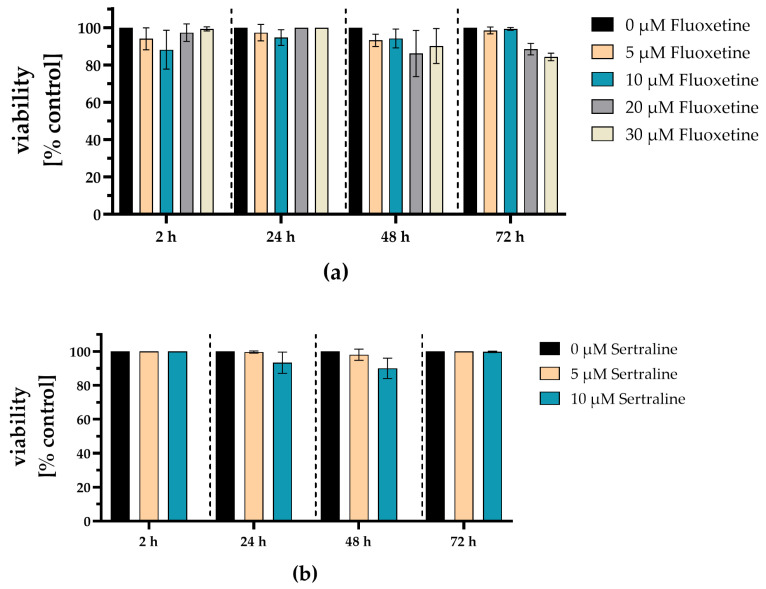
Fluoxetine and sertraline showed no toxic effects on A549-AT cells. Potential cytotoxic effects of fluoxetine or sertraline were tested towards A549-AT cells using “Orangu cell counting solution”. Different concentrations of (**a**) fluoxetine or (**b**) sertraline were incubated with a confluent layer of A549-AT cells and the cell viability was evaluated after 2, 24, 48 and 72 h. All experiments were performed as triplicate. Data are shown as mean and standard deviation (SD). Differences between fluoxetine/sertraline and the control were compared by one-way ANOVA.

Potential cytotoxic effects of the antidepressants fluoxetine and sertraline were analyzed by determining cell viability. Therefore, A549-AT cells were incubated with different concentrations of fluoxetine (0–30 µM) or sertraline (0–10 µM) and tested for viability after 2, 24, 48 and 72 h of incubation at 37 °C and 5% CO_2_. After 72 h, no substantial toxicity could be detected even if the cells were exposed to the highest fluoxetine (30 µM) or sertraline (10 µM) concentrations. Thus, fluoxetine and sertraline potently neutralize SARS-CoV-2 D614G and the variants alpha, delta, omicron BA.1 and omicron BA5 at subtoxic concentrations (Figure 7).

## 4. Discussion

After three years of the pandemic, SARS-CoV-2 remains a global health burden marked by the persistence of severe courses of disease with substantial numbers of hospitalizations and deaths. Furthermore, in many cases, there are long-lasting symptoms, known as long-COVID-19. With increasing vaccination fatigue and a growing number of vaccine-breakthrough infections, there is an urgent need for rapidly available drugs to treat or prevent SARS-CoV-2 infections, in particular such with newly emerging variants. Various antidepressants were described as potent entry inhibitors of early-emerged SARS-CoV-2 variants and other coronaviruses [17,18]. However, it remained unclear whether antidepressants that target the SARS-CoV-2 spike S1 domain may inhibit newly emerging SARS-CoV-2 variants despite mutations that additionally occur during SARS-CoV-2 evolution.

In the present study, we investigated the antiviral efficacy of fluoxetine and sertraline against distinct SARS-CoV-2 variants, including D614G, alpha, delta, omicron BA.1 and omicron BA.5. As a first step, the ability of fluoxetine and sertraline to inhibit SARS-CoV-2 infection was examined by using VSV*ΔG-Fluc spike pseudotyped virus-like particles with mutations within the RBD or the full spike S1 domain. This method offers the advantage of rapid testing of active substances against different SARS-CoV-2 variants without the need for an S3 laboratory [35,36]. In line with previous studies, fluoxetine and sertraline effectively blocked the infection with VSV*ΔG-Fluc spike pseudotyped virus-like particles [7,37]. The antiviral activity of fluoxetine and sertraline was shown for the first time on this broad variety of variants.

Notably, fluoxetine and sertraline completely neutralized the replication of all used clinical SARS-CoV-2 isolates (D614G, alpha, delta, omicron BA.1 and omicron BA.5) at a subtoxic concentration of 30 µM (fluoxetine) or 10 µM (sertraline). The neutralizing effect of these antidepressants was unaffected by the respective mutations within the SARS-CoV-2 spike protein. This is an interesting finding, as fluoxetine and sertraline were described to interfere with SARS-CoV-2 entry, and at least sertraline directly targeted the SARS-CoV-2 spike protein [7]. In addition to targeting the spike protein, fluoxetine and sertraline were described as functional inhibitors of the acid sphingomyelinase (ASM), which plays an important role during SARS-CoV-2 entry into the host cell [38,39]. ASM is a glycoprotein that catalyzes sphingomyelin degradation to phosphorylcholine and ceramide, which is known to facilitate viral entry into the host cell [40,41,42]. SARS-CoV-2 activates the ASM/ceramide system, resulting in the formation of ceramide-enriched membrane domains that cluster ACE2 and thereby facilitate viral entry and infection [39,40,43]. Thus, inhibiting ASM represents a SARS-CoV-2 spike mutation-independent mechanism to interfere with viral entry and infection. In the context of other viral infections, our data indicate that FIASMAs are effective not only against, for example, rhinoviruses [44] or ebolaviruses [45], but also against SARS-CoV-2. In line with prior studies, our findings suggest that fluoxetine and sertraline use in early-stage SARS-CoV-2 infections with recent variants may reduce the risk of a severe course of COVID-19 or death [46].

However, we have demonstrated the antiviral efficacy of fluoxetine and sertraline against the respective SARS-CoV-2 variants in cell culture. Nonetheless, deriving the exact dosage for humans solely from cell culture experiments is not feasible, as there are additional crucial factors such as the immune system involved in fighting the infection in humans. Determining the effective dosage for human intervention is the subject of ongoing clinical studies.

Randomized clinical trials and retrospective studies have already demonstrated that treatment with antidepressants result in a milder course of COVID-19 and a reduced mortality rate [20,23,47,48,49,50]. Furthermore, there was a lower infection rate among patients using antidepressants, indicating that fluoxetine and sertraline may have a protective effect against SARS-CoV-2 infections [51].

In conclusion, the antidepressants fluoxetine and sertraline are promising candidates for the supportive treatment of SARS-CoV-2 infections, in particular during the early phase of infection. Especially in developing countries with a restricted medical health care system, these cheap and generally available drugs may contribute to improve the treatment of COVID-19 patients. Since their antiviral efficacy is unaffected by SARS-CoV-2 spike mutations, they may be used in new or even breakthrough infections with newly emerging SARS-CoV-2 variants.

## Data Availability

The data presented in this study are available on request from the corresponding author.

## Supplementary Materials

The following supporting information can be downloaded at: https://www.mdpi.com/article/10.3390/v16040545/s1, Table S1: Primers used for mutagenesis and sequencing; Table S2: Mutations in respective plasmids and corresponding SARS-CoV-2 variant; Table S3: M- and N-gene-specific primers used for RT-qPCR.

## References

[1] Worldometers. https://www.worldometers.info/coronavirus/.

[2] Panneer S., Kantamaneni K., Akkayasamy V.S., Susairaj A.X., Panda P.K., Acharya S.S., Rice L., Liyanage C., Pushparaj R.R.B. (2022). The Great Lockdown in the Wake of COVID-19 and Its Implications: Lessons for Low and Middle-Income Countries. Int. J. Environ. Res. Public Health.

[3] Stamm T.A., Partheymüller J., Mosor E., Ritschl V., Kritzinger S., Alunno A., Eberl J.-M. (2023). Determinants of COVID-19 vaccine fatigue. Nat. Med..

[4] Bormann M., Brochhagen L., Alt M., Otte M., Thümmler L., van de Sand L., Kraiselburd I., Thomas A., Gosch J., Braß P. (2023). Immune responses in COVID-19 patients during breakthrough infection with SARS-CoV-2 variants Delta, Omicron-BA.1 and Omicron-BA.5. Front. Immunol..

[5] Bergwerk M., Gonen T., Lustig Y., Amit S., Lipsitch M., Cohen C., Mandelboim M., Levin E.G., Rubin C., Indenbaum V. (2021). Covid-19 Breakthrough Infections in Vaccinated Health Care Workers. N. Engl. J. Med..

[6] Dash N.R., Barqawi H.J., Obaideen A.A., Al Chame H.Q., Samara K.A., Qadri R., Eldesouki S. (2023). COVID-19 Breakthrough Infection Among Vaccinated Population in the United Arab Emirates. J. Epidemiol. Glob. Health.

[7] Chen Y., Wu Y., Chen S., Zhan Q., Wu D., Yang C., He X., Qiu M., Zhang N., Li Z. (2022). Sertraline Is an Effective SARS-CoV-2 Entry Inhibitor Targeting the Spike Protein. J. Virol..

[8] Manganaro R., Zonsics B., Bauer L., Lopez M.L., Donselaar T., Zwaagstra M., Saporito F., Ferla S., Strating J.R.P.M., Coutard B. (2020). Synthesis and antiviral effect of novel fluoxetine analogues as enterovirus 2C inhibitors. Antivir. Res..

[9] Messacar K., Sillau S., Hopkins S.E., Otten C., Wilson-Murphy M., Wong B., Santoro J.D., Treister A., Bains H.K., Torres A. (2019). Safety, tolerability, and efficacy of fluoxetine as an antiviral for acute flaccid myelitis. Neurology.

[10] Tseng K.C., Hsu B.Y., Ling P., Lu W.W., Lin C.W., Kung S.H. (2022). Antidepressant Sertraline Is a Broad-Spectrum Inhibitor of Enteroviruses Targeting Viral Entry through Neutralization of Endolysosomal Acidification. Viruses.

[11] Zuo J., Quinn K.K., Kye S., Cooper P., Damoiseaux R., Krogstad P. (2012). Fluoxetine Is a Potent Inhibitor of Coxsackievirus Replication. Antimicrob. Agents Chemother..

[12] Patel K., Lim S.G., Cheng C.W., Lawitz E., Tillmann H.L., Chopra N., Altmeyer R., Randle J.C.R., McHutchison J.G. (2011). Open-label Phase 1b pilot study to assess the antiviral efficacy of simvastatin combined with sertraline in chronic hepatitis C patients. Antivir. Ther..

[13] Herring S., Oda J.M., Wagoner J., Kirchmeier D., O’Connor A., Nelson E.A., Huang Q.F., Liang Y.Y., DeWald L.E., Johansen L.M. (2021). Inhibition of Arenaviruses by Combinations of Orally Available Approved Drugs. Antimicrob. Agents Chemother..

[14] Albouz S., Boutry J.M., Dubois G., Bourdon R., Hauw J.J., Baumann N. (1981). Lipid and lysosomal enzymes in human fibroblasts cultured with perhexiline maleate. Naunyn-Schmiedebergs Arch. Pharmacol..

[15] Hurwitz R., Ferlinz K., Sandhoff K. (1994). The Tricyclic Antidepressant Desipramine Causes Proteolytic Degradation of Lysosomal Sphingomyelinase in Human Fibroblasts. Biol. Chem. Hoppe-Seyler.

[16] Kornhuber J., Tripal P., Reichel M., Mühle C., Rhein C., Muehlbacher M., Groemer T.W., Gulbins E. (2010). Functional Inhibitors of Acid Sphingomyelinase (FIASMAs): A novel pharmacological group of drugs with broad clinical applications. Cell Physiol. Biochem..

[17] Zimniak M., Kirschner L., Hilpert H., Geiger N., Danov O., Oberwinkler H., Steinke M., Sewald K., Seibel J., Bodem J. (2021). The serotonin reuptake inhibitor Fluoxetine inhibits SARS-CoV-2 in human lung tissue. Sci. Rep..

[18] Kutkat O., Moatasim Y., Al-Karmalawy A.A., Abulkhair H.S., Gomaa M.R., El-Taweel A.N., Abo Shama N.M., GabAllah M., Mahmoud D.B., Kayali G. (2022). Robust antiviral activity of commonly prescribed antidepressants against emerging coronaviruses: In vitro and in silico drug repurposing studies. Sci. Rep..

[19] Péricat D., Leon-Icaza S.A., Sanchez Rico M., Mühle C., Zoicas I., Schumacher F., Planès R., Mazars R., Gros G., Carpinteiro A. (2022). Antiviral and Anti-Inflammatory Activities of Fluoxetine in a SARS-CoV-2 Infection Mouse Model. Int. J. Mol. Sci..

[20] Lenze E.J., Mattar C., Zorumski C.F., Stevens A., Schweiger J., Nicol G.E., Miller J.P., Yang L., Yingling M., Avidan M.S. (2020). Fluvoxamine vs Placebo and Clinical Deterioration in Outpatients with Symptomatic COVID-19: A Randomized Clinical Trial. JAMA.

[21] Calusic M., Marcec R., Luksa L., Jurkovic I., Kovac N., Mihaljevic S., Likic R. (2022). Safety and efficacy of fluvoxamine in COVID-19 ICU patients: An open label, prospective cohort trial with matched controls. Br. J. Clin. Pharmacol..

[22] Seftel D., Boulware D.R. (2021). Prospective Cohort of Fluvoxamine for Early Treatment of Coronavirus Disease 19. Open Forum. Infect. Dis..

[23] Reis G., Silva E., Silva D.C.M., Thabane L., Milagres A.C., Ferreira T.S., Dos Santos C.V.Q., Campos V.H.S., Nogueira A.M.R., de Almeida A. (2022). Effect of Early Treatment with Ivermectin among Patients with COVID-19. N. Engl. J. Med..

[24] Carpinteiro A., Edwards M.J., Hoffmann M., Kochs G., Gripp B., Weigang S., Adams C., Carpinteiro E., Gulbins A., Keitsch S. (2020). Pharmacological Inhibition of Acid Sphingomyelinase Prevents Uptake of SARS-CoV-2 by Epithelial Cells. Cell Rep. Med..

[25] Carpinteiro A., Gripp B., Hoffmann M., Pöhlmann S., Hoertel N., Edwards M.J., Kamler M., Kornhuber J., Becker K.A., Gulbins E. (2021). Inhibition of acid sphingomyelinase by ambroxol prevents SARS-CoV-2 entry into epithelial cells. J. Biol. Chem..

[26] Hoertel N. (2021). Do the Selective Serotonin Reuptake Inhibitor Antidepressants Fluoxetine and Fluvoxamine Reduce Mortality among Patients with COVID-19?. JAMA Netw. Open.

[27] Hoertel N., Sánchez-Rico M., Cougoule C., Gulbins E., Kornhuber J., Carpinteiro A., Becker K.A., Reiersen A.M., Lenze E.J., Seftel D. (2021). Repurposing antidepressants inhibiting the sphingomyelinase acid/ceramide system against COVID-19: Current evidence and potential mechanisms. Mol. Psychiatry.

[28] Widera M., Wilhelm A., Toptan T., Raffel J.M., Kowarz E., Roesmann F., Grozinger F., Siemund A.L., Luciano V., Kulp M. (2021). Generation of a Sleeping Beauty Transposon-Based Cellular System for Rapid and Sensitive Screening for Compounds and Cellular Factors Limiting SARS-CoV-2 Replication. Front. Microbiol..

[29] Thümmler L., Gäckler A., Bormann M., Ciesek S., Widera M., Rohn H., Fisenkci N., Otte M., Alt M., Dittmer U. (2022). Cellular and Humoral Immunity against Different SARS-CoV-2 Variants Is Detectable but Reduced in Vaccinated Kidney Transplant Patients. Vaccines.

[30] WHO. https://www.who.int/activities/tracking-SARS-CoV-2-variants.

[31] Heilingloh C.S., Aufderhorst U.W., Schipper L., Dittmer U., Witzke O., Yang D., Zheng X., Sutter K., Trilling M., Alt M. (2020). Susceptibility of SARS-CoV-2 to UV irradiation. Am. J. Infect. Control.

[32] Hoffmann M., Kleine-Weber H., Schroeder S., Krüger N., Herrler T., Erichsen S., Schiergens T.S., Herrler G., Wu N.H., Nitsche A. (2020). SARS-CoV-2 Cell Entry Depends on ACE2 and TMPRSS2 and Is Blocked by a Clinically Proven Protease Inhibitor. Cell.

[33] Berger Rentsch M., Zimmer G. (2011). A vesicular stomatitis virus replicon-based bioassay for the rapid and sensitive determination of multi-species type I interferon. PLoS ONE.

[34] Becker K.A., Carpinteiro A., Hoffmann M., Pöhlmann S., Kornhuber J., Gulbins E. (2021). Ex vivo assay to evaluate the efficacy of drugs targeting sphingolipids in preventing SARS-CoV-2 infection of nasal epithelial cells. STAR Protoc..

[35] Zettl F., Meister T.L., Vollmer T., Fischer B., Steinmann J., Krawczyk A., V’kovski P., Todt D., Steinmann E., Pfaender S. (2020). Rapid Quantification of SARS-CoV-2-Neutralizing Antibodies Using Propagation-Defective Vesicular Stomatitis Virus Pseudotypes. Vaccines.

[36] Hoffmann M., Hofmann-Winkler H., Krüger N., Kempf A., Nehlmeier I., Graichen L., Arora P., Sidarovich A., Moldenhauer A.S., Winkler M.S. (2021). SARS-CoV-2 variant B.1.617 is resistant to bamlanivimab and evades antibodies induced by infection and vaccination. Cell Rep..

[37] Fred S.M., Kuivanen S., Ugurlu H., Casarotto P.C., Levanov L., Saksela K., Vapalahti O., Castrén E. (2021). Antidepressant and Antipsychotic Drugs Reduce Viral Infection by SARS-CoV-2 and Fluoxetine Shows Antiviral Activity Against the Novel Variants in vitro. Front. Pharmacol..

[38] Geiger N., Kersting L., Schlegel J., Stelz L., Fähr S., Diesendorf V., Roll V., Sostmann M., König E.-M., Reinhard S. (2022). The Acid Ceramidase Is a SARS-CoV-2 Host Factor. Cells.

[39] Schloer S., Brunotte L., Goretzko J., Mecate-Zambrano A., Korthals N., Gerke V., Ludwig S., Rescher U. (2020). Targeting the endolysosomal host-SARS-CoV-2 interface by clinically licensed functional inhibitors of acid sphingomyelinase (FIASMA) including the antidepressant fluoxetine. Emerg. Microbes Infect..

[40] Kornhuber J., Hoertel N., Gulbins E. (2022). The acid sphingomyelinase/ceramide system in COVID-19. Mol. Psychiatry.

[41] Törnquist K., Asghar M.Y., Srinivasan V., Korhonen L., Lindholm D. (2021). Sphingolipids as Modulators of SARS-CoV-2 Infection. Front. Cell Dev. Biol..

[42] Beckmann N., Becker K.A. (2021). Ceramide and Related Molecules in Viral Infections. Int. J. Mol. Sci..

[43] Hashimoto Y., Suzuki T., Hashimoto K. (2022). Mechanisms of action of fluvoxamine for COVID-19: A historical review. Mol. Psychiatry.

[44] Grassme H., Riehle A., Wilker B., Gulbins E. (2005). Rhinoviruses infect human epithelial cells via ceramide-enriched membrane platforms. J. Biol. Chem..

[45] Miller M.E., Adhikary S., Kolokoltsov A.A., Davey R.A. (2012). Ebolavirus requires acid sphingomyelinase activity and plasma membrane sphingomyelin for infection. J. Virol..

[46] Hoertel N., Rezaei K., Sánchez-Rico M., Delgado-Álvarez A., Kornhuber J., Gulbins E., Olfson M., Ouazana-Vedrines C., Carpinteiro A., Cougoule C. (2023). Medications Modulating the Acid Sphingomyelinase/Ceramide System and 28-Day Mortality among Patients with SARS-CoV-2: An Observational Study. Pharmaceuticals.

[47] Reis G., Dos Santos Moreira Silva E.A., Medeiros Silva D.C., Thabane L., de Souza Campos V.H., Ferreira T.S., Quirino Dos Santos C.V., Ribeiro Nogueira A.M., Figueiredo Guimaraes Almeida A.P., Cançado Monteiro Savassi L. (2023). Oral Fluvoxamine with Inhaled Budesonide for Treatment of Early-Onset COVID-19: A Randomized Platform Trial. Ann. Intern. Med..

[48] Wang H., Wei Y., Hung C.T., Jiang X., Li C., Jia K.M., Leung E.Y.M., Yam C.H.K., Chow T.Y., Zhao S. (2023). Relationship between antidepressants and severity of SARS-CoV-2 Omicron infection: A retrospective cohort study using real-world data. Lancet Reg. Health West. Pac..

[49] Jittamala P., Boyd S., Schilling W.H., Watson J.A., Ngamprasertchai T., Siripoon T., Luvira V., Batty E.M., Wongnak P., Esper L.M. (2024). Antiviral efficacy of fluoxetine in early symptomatic COVID-19: An open-label, randomised, controlled, adaptive platform trial (PLATCOV). medRxiv.

[50] Reis G., Dos Santos Moreira-Silva E.A., Silva D.C.M., Thabane L., Milagres A.C., Ferreira T.S., Dos Santos C.V.Q., de Souza Campos V.H., Nogueira A.M.R., de Almeida A. (2022). Effect of early treatment with fluvoxamine on risk of emergency care and hospitalisation among patients with COVID-19: The TOGETHER randomised, platform clinical trial. Lancet Glob. Health.

[51] Oskotsky T., Maric I., Tang A., Oskotsky B., Wong R.J., Aghaeepour N., Sirota M., Stevenson D.K. (2021). Mortality Risk Among Patients with COVID-19 Prescribed Selective Serotonin Reuptake Inhibitor Antidepressants. JAMA Netw Open.

