# Supplementary material for: Fluoxetine and Sertraline Potently Neutralize the Replication of Distinct SARS-CoV-2 Variants
Source: Viruses. 2024 Mar 30;16(4):545. doi: 10.3390/v16040545 (PMC11053511; doi:10.3390/v16040545)
Supplement: Supplementary file 1 [file viruses-16-00545-s001.zip › viruses-2896435-supplementary.pdf]

Table S1: Primers used for mutagenesis and sequencing.

Mutation	Mutagenesis primers	Sequencing primers
R346K	Fw: AATGCCACCA**a**ATTCGCCTCTG<br>Rev: GAACACCTCGCCGAAGGG	Fw:<br>GTGGATTGTGCCCTTGATCCTC
K417N	Fw: AGACAGGCAA**c**ATCGCCGACTAC<br>Rev: GTCCAGGGGCAATCTGCG	Fw: CAGCTTCAGCACCTTCAAG
K417T	Fw: CAGACAGGCA**c**GATCGCCGAC<br>Rev: TCCAGGGGCAATCTGCCG	Fw: CAGCTTCAGCACCTTCAAG
L452Q	Fw: TACAATTACC**a**GTACCGGCTGTTCC<br>Rev: GTTGCCGCCGACTTTGGA	Fw: CAGCTTCAGCACCTTCAAG
L452R	Fw: TACAATTACC**g**GTACCGGCTGTTC<br>Rev: GTTGCCGCCGACTTTGGA	Fw: CAGCTTCAGCACCTTCAAG
T478K	Fw: GCCGGCAGCA**aa**CCTTGTAACG<br>Rev: CTGATAGATCTCGGTGGAG	Fw: CAGCTTCAGCACCTTCAAG
E484K	Fw:<br>TAACGGCGTG**a**AAGGCTTCAACTGCTAC<br>Rev: CAAGGGGTGCTGCCGGCC	Fw: CAGCTTCAGCACCTTCAAG
F490S	Fw: AACTGCTACT**c**CCCACTGCAG<br>Rev: GAAGCCTTCCACGCCGTT	Fw: CAGCTTCAGCACCTTCAAG
N501Y	Fw: TCAGCCCACA**t**ATGGCGTGGG<br>Rev: AAGCCGTAGGACTGCAGTG	Fw: CAGCTTCAGCACCTTCAAG

Table S2. Mutations in respective plasmids and corresponding SARS-CoV-2 variant.

Plasmid	RBD mutations	Non-RBD mutations	Corresponding SARS-CoV-2 variant(s)
pCG1-SARS-S-del18	-	18aa deletion in c-terminal tail	Wuhan-Hu-1<br>(Wt)
pCG1-SARS-S-del18_N501Y	N501Y	18aa deletion in c-terminal tail	B.1.1.7<br>(alpha) – RBD only
pCG1-SARS-S-del18_K417N-E484K-N501Y	K417N, E484K, N501Y	18aa deletion in c-terminal tail	B.1.351<br>(beta) – RBD only
pCG1-SARS-S-del18_K417T-E484K-N501Y	K417N, E484K, N501Y	18aa deletion in c-terminal tail	P.1<br>(gamma) – RBD only
pCG1-SARS-S-del18_L452R-T478K	L452R, T478K	18aa deletion in c-terminal tail	B.1.617.2<br>(delta) – RBD only
pCG1-SARS-S-del18_L452Q-F490S	L452Q, F490S	18aa deletion in c-terminal tail	C.37<br>(lambda) – RBD only
pCG1-SARS-S-del18_R346K-E484K-N501Y	R346K, E484K, N501Y	18aa deletion in c-terminal tail	B.1.621<br>(mu) – RBD only
pCDNA3.3_CoV2_D18	-	18aa deletion in c-terminal tail	Wuhan-Hu-1<br>(Wt)
pCDNA3.3_CoV2_B.1.1.7	N501Y	69-70del, 144del, A570D, D614G, P681H, T716I, S982A, D1118H, 18aa deletion in c-terminal tail	B.1.1.7<br>(alpha)

pCDNA3.3_CoV2_501V2	K417N, E484K, N501Y	L18F, D80A, D215G, R246I, D614G, A7801V, 18aa deletion in c-terminal tail	B.1.351 (beta)
pCDNA3.3_CoV2_P1	K417N, E484K, N501Y	L18F, T20N, P26S, D138Y, R190S, D614G, H655Y, T1027I, 18aa deletion in c-terminal tail	P.1 (gamma)
pCDNA3.3-SARS2-B.1.617.2	L452R, T478K	T19R, 156G, 157-158del, D614G, P681R, D950N, 18aa deletion in c-terminal tail	B.1.617.2 (delta)
SARS-CoV-2 Omicron Strain S gene Human codon_pcDNA3.1(+)	G339D, S371L, S373P, S375F, K417N, N440K, G446S, S477N, T478K, E484A, Q493R, G498R, N501Y, Y505H	A67V, 67-68del, T95I, G142D, 143-145del, N211I, 212del, ins215EPE, T547K, D614G, H655Y, N679K, P681H, N764K, D796Y, N856K, Q954H, N969K, L918F, 18aa deletion in c-terminal tail	B.1.1.529 (omicron)

Table S3. M- and N-gene-specific primers used for RT-qPCR.

Primer	Sequence (5´- 3´)
M-gene fwd	tgtgacatcaaggacctgcc
M-gene rev	ctgagtcacctgctacacgc
M-gene probe (PMID 32575728)	FAM-tgttgctacatcacgaacgc-BHQ1
N-gene fwd (pWhSF-N-F9)	acattggcacccgcaatc
N-gene rev (pWhSF-N-R10)	cgagaagaggcttgactgcc
N-gene probe (pWhSF-N-P11lna)	FAM-cct+caaggaa+caa+catt-BHQ1

(+ = LNA (locked nucleic acid) modified)
